# Implementation and effectiveness of 'care navigation', coordinated management for people with complex chronic illness: rationale and methods of a randomised controlled

**DOI:** 10.1186/1472-6963-13-164

**Published:** 2013-05-03

**Authors:** Natalie Plant, Kylie-Ann Mallitt, Patrick J Kelly, Tim Usherwood, James Gillespie, Steven Boyages, Stephen Jan, Justin McNab, Beverley M Essue, Kathy Gradidge, Nereus Maranan, David Ralphs, Clive Aspin, Stephen Leeder

**Affiliations:** 1 Menzies Centre for Health Policy, University of Sydney, Sydney, NSW, Australia; 2 Sydney School of Public Health, University of Sydney, Sydney, NSW, Australia; 3 Discipline of General Practice, University of Sydney, Sydney, NSW, Australia; 4 eHealthNSW, NSW Health, Sydney, NSW, Australia; 5 The George Institute for Global Health, Sydney, NSW, Australia; 6 Patient Flow Unit, Nepean Hospital, Sydney, NSW, Australia

**Keywords:** Randomised controlled trial, Chronic illness, Comorbidity, Hospital admissions, Community health, Hospital length of stay, Coordinated care, Process evaluation

## Abstract

**Background:**

Chronic illness is a significant driver of the global burden of disease and associated health care costs. People living with severe chronic illness are heavy users of acute hospital services; better coordination of their care could potentially improve health outcomes while reducing hospital use. The Care Navigation trial will evaluate an in-hospital coordinated care intervention on health service use and quality of life in chronically ill patients.

**Methods/Design:**

A randomised controlled trial in 500 chronically ill patients presenting to the emergency department of a hospital in Western Sydney, Australia. Participants have three or more hospital admissions within a previous 12 month period and either aged ≥70 years; or aged ≥45 years and of Aboriginal or Torres Strait Islander descent; or aged ≥ 16 with a diagnosis of a respiratory or cardiology related illness. Patients are randomised to either the coordinated care program (Care Navigation), or to usual care. The Care Navigation program consists of dedicated nurses who conduct patient risk assessments, oversee patient nursing while in hospital, and guide development of a care plan for the management of chronic illness after being discharged from hospital. These nurses also book community appointments and liaise with general practitioners. The main outcome variables are the number of emergency department re-presentations and hospital readmissions, and quality of life during a 24 month follow-up. Secondary outcomes are length of hospital stay, mortality, time to first hospital re-admission, time to first emergency department re-presentation, patient satisfaction, adherence to prescribed medications, amount and type of in-hospital referrals made for consultations and diagnostic testing, and the number and type of community health referrals. A process evaluation and economic analysis will be conducted alongside the randomised trial.

**Discussion:**

A trial of in-hospital care coordination may support recent evidence that engaging primary health services in care plans linked to multidisciplinary team support improves patient outcomes and reduces costs to the health system. This will inform local, national and international health policy.

**Trial registration:**

Australia New Zealand Clinical Trials Registry ACTRN12609000554268

## Background

Chronic illness is a significant contributor to the global burden of disease, with markedly increasing prevalence and associated stress on health care systems [1]. In Australia, 80% of the estimated total burden of disease is attributed to chronic illness, primarily cardiovascular disease, chronic respiratory conditions, diabetes and cancer [2].

Population ageing exacerbates the burden of chronic disease in developed countries [2]. Health service needs for older populations are complex, involving multiple comorbidities and a broader range of service providers [3]. It is estimated that among independently living Australians, almost half aged 65–74 have five or more chronic conditions, increasing to 70% of those aged 85 and over [4].

People with multiple chronic illnesses are major consumers of health care services [5], accounting for 70% of general practice consultations. They are twice as likely to be admitted to hospital, and stay in hospital disproportionately longer [2]. The substantial cost to government is further challenged by the need to strengthen disease prevention initiatives while providing care to a growing number of patients with chronic disease [6].

High health care costs are driven by the episodic nature of standard health services, which are focused around acute care. This model may be poorly structured and unsupported in the context of patients with chronic illness who can require multiple presentations across private medical specialists, community health, general practice, allied health, and hospital inpatient and outpatient clinics. International research over the past decade suggests that chronic illness may be more effectively treated by better connecting patients’ health management from hospital to community-based services [2,3,5,7-12]. Coordinated care has been identified as a solution which provides this link.

The aim of coordinated (or integrated) care is to enhance quality of care and quality of life, consumer satisfaction and system efficiency for patients with complex, long-term problems across multiple services, providers and settings [13]. Coordinated care can take on a variety of forms, but always involves multidisciplinary communication and care planning [9,14]. Trials to assess the effectiveness of coordinated care have been conducted across the OECD, including in Australia, USA, Canada, England, Italy, Denmark and France [2,3,5,7-12].

A positive effect of community-based care has generally been shown in trials, with lower rates of hospitalisation and lower costs [3,8,10]. Improvements in the level of service access and patient knowledge have also been observed [2]. However, there is inadequate evaluation of the outcomes, and particularly the implementation, of coordinated care interventions within health services. Within the Australian setting, a randomised controlled trial (RCT) of the outcomes of face-to-face coordinated care has not been conducted [2]. More broadly, trials are often focused on specific conditions or population subgroups, and are conducted with small sample sizes and over short timeframes. In addition, trials conducted to date employ a highly variable degree of service coordination.

Solid evidence is required of the effectiveness of care coordination to improve patient outcomes in the population. Implementation of care programs must be evaluated in a way that allows health services to gauge which of its components (e.g., emergency department, wards, discharge, community practitioners) and which of its stakeholders (e.g., patients, carers, nurses, doctors, management, policymakers) facilitate or create barriers to the effectiveness of the new interventions.

### Study objectives

A randomised control trial (RCT) will be conducted to measure the impact of an in-hospital coordinated care intervention, Care Navigation (CN), on health service use and quality of life in elderly and chronically ill patients in Western Sydney, Australia. Effectiveness is based on emergency hospital presentations, hospital admissions and quality of life over 24 months. An economic evaluation examines the cost-effectiveness of CN against current standard care from the perspective of the health sector. A process evaluation will also be conducted to describe the organisational setting and context within which CN has been developed and implemented.

## Methods/Design

### Design and study site

The CN study is a single-blind pragmatic RCT of chronically ill patients presenting to the emergency department of Nepean Hospital. Patients are randomly assigned to receive CN (intervention arm) or standard care (control arm). Recruitment is conducted over 10 months, and the two trial arms are followed for 24 months.

Nepean Hospital is a tertiary teaching hospital in Western Sydney, Australia. It provides inpatient and ambulatory services, and averages 580 overnight beds. In 2009–2010 it provided 28,989 overnight admissions and 19,113 day-only admissions. There were 16,562 medical emergency admissions in that year and a total of 51,414 presentations to the emergency department [15].

### Study participants

The study includes patients identified by an inclusion algorithm implemented within the patient tracking system of the emergency department, or by clinician recommendation. The inclusion algorithm identifies patients who have three or more admissions to a Sydney West Area Health Service hospital within a previous 12 month period and are either aged ≥70 years; or aged ≥45 years and of Aboriginal or Torres Strait Islander descent; or aged ≥ 16 with at least one admission for a respiratory or cardiology related condition. Patients are also eligible if a treating clinician determines that a patient would benefit from receiving CN.

Exclusion criteria are not providing written informed consent; physical or mental inability to give consent or participate in follow-up activities (e.g. dementia); previously received CN; or where language skills prevent questionnaire completion.

### Consent and randomisation

Written informed consent is obtained from all patients. All patients identified by the inclusion screening process are either a) approached in person by a research officer or CN nurse to discuss study participation; or b) approached by telephone, if the patient is identified by the screening process out of hours. All patients approached by telephone are given a patient information sheet while in the hospital, and sign a ‘consent to approach’ form, giving permission for study staff to contact them by telephone.

Randomisation is 1:1 allocation and is conducted using permuted block design and stratified by age group (age ≥ 70, or age ≥ 45 for Aboriginal or Torres Strait islander patients). The randomisation is administered by an independent body (the NHMRC Clinical Trials Centre’s Randomisation Service), who are contacted by telephone for the allocation of each patient to a study arm. Allocation remains masked until after recruitment to the study. Once a participant is randomised, the research officer, the CN nurses and participant are not blinded. The research assistant collecting participant follow-up data remains blind to the treatment allocation throughout the two-year follow-up period. Study statisticians remain blind to allocation until after statistical analyses are complete.

### Description of the intervention

a) Control arm: current standard care

Patients randomised to current standard care are directed through the hospital system according to the symptoms they exhibit on presentation to the emergency department. This may involve treatment in the emergency department only, or require hospitalisation before discharge. Patients could be linked with hospital and community-based health care facilities, as may normally occur in the absence of CN.

b) Intervention arm: Care Navigation

Care Navigation involves Inbound, Inflight and Outbound care components. The *Inbound* CN nurse receives an electronic page and on-screen alert advising them of an automated referral for a CN consultation at the time of a patient’s presentation to the emergency department. A risk assessment tool to assess the patient’s risk of re-presentation is performed, and a profile of that patient’s health needs is developed and stored electronically, incorporating the patient’s hospital and community health based record, and the patient’s input. Using this profile, the Inbound CN nurse collaborates with emergency medical officers to determine whether the patient requires admission to hospital, or community-based health care. The *Inflight* CN nurse continues patient care after hospital admission. They oversee patient nursing and guide development of a care plan for the management of chronic illness after being discharged from hospital. Patients are care coordinated and/or case managed by the *Outbound* CN nurse, either from the ward or from the emergency department. The Outbound CN nurse collaborates with the patient’s multidisciplinary care team to ensure the patient is ready and safe for discharge, develops community-based care plans, books appointments and contacts general practitioners (GPs). The Outbound CN nurse also makes follow-up phone calls to patients. Care plans are recorded in the patient’s electronic hospital record, faxed to the patients GP, and are revised and updated as required at any subsequent acute hospital presentations and admissions.

### Study variables

In addition to the inclusion and exclusion criteria, six types of variables are collected: demographic and other risk factors, psychosocial, hospital utilisation, community health utilisation, medication usage, and mortality variables. Outcome variables are calculated after 12 months and/or 24 months of follow-up period (Table 1).

**Table 1:** Quantitative data collected in the Care Navigation randomised controlled trial

**Type of data**	**Variables**	**Data source**	**Time of data collection**
**Demographic and risk factor variables**	Age, sex, Indigenous status, marital status	Electronic report from NSW Health database	Baseline.
	Literacy, language, ethnicity, income, education, BMI, living arrangements smoking, alcohol, physical and mental disability, comorbidities	Phone questionnaire-manual data entry	12 months.
**Psychosocial variables**	Quality of life: EQ-5D questionnaire [16]	Phone questionnaire-manual data entry	Baseline, 12 months, 24 months.
	Patient experience: Picker patient experience questionnaire [18]	Mailed paper questionnaire-manual data entry	12 months.
**Hospital utilisation variables**	*For each emergency presentation within the follow-up period:* Triage category, arrival date and time, departure date and time, mode of separation, presenting problem and diagnosis, UDAG status	Electronic report from NSW Health database	24 months.
	*For each hospital admission within the follow-up period:* Arrival date and time, departure date and time, diagnosis related group (DRG), service related group (SRG), investigations conducted	Electronic report from NSW Health database	24 months.
**Community health utilisation variables**	*For each community referral within the follow-up period:* Referral service type, appointment date and time, treatment administered, attendance and transport	Electronic report from NSW Health database	24 months.
**Medication utilisation variables**	Clinical service utilisation (MBS)	Electronic report from Statistics Medicare Australia	24 months.
	Pharmaceutical utilisation (PBS)	Phone questionnaire (manual data entry)	Baseline, 12 months, 24 months.
	Adherence to medications [19]		
**Mortality data**	Date of death	Electronic report from the National Death Index (Australian Institute of Health and Welfare)	24 months.

The three primary outcome measures are a) the rate of re-presentations* to the emergency department; b) the rate of hospital re-admissions*, and c) quality of life. Secondary outcome measures are the length of hospital stay, mortality rates, time to first hospital re-admission, time to first ED re-presentation, hospital key performance indicators, patient satisfaction, and adherence to prescribed medications.

*Includes presentations and admissions to any Western Sydney or Nepean Blue Mountains Local Health District hospital.

### Study size

A total of 500 patients are recruited for this study. With an expected 20% loss to follow-up, this results in a final sample size of 400, giving over 90% power to detect a 20% reduction in hospital admissions at a 5% significance level. This effect size is a rate ratio of 0.8 based on an average of 2.5 admissions per patient over a two year period in the control group, compared to 2.0 in the intervention group. The study has more than 80% power to detect a clinically significant difference in ED re-presentations, since ED presentations are more common than hospital admissions. A sample size of 400 would also allow us to detect a mean difference of 10 points on the EQ-5D scale [16], with approximately 80% power at a 5% significance level.

### Statistical analysis

Cox proportional hazards models are used to analyse time to first ED presentation, time to first readmission and time to mortality. Readmission rates and length of stay are analysed using Poisson or Negative Binomial models. EQ-5D is analysed using analysis of variance, and t-tests for pairwise comparisons. Baseline characteristics, such as age, gender and severity of illness are examined. Model checking of assumptions and good-of fit tests are conducted for all analyses and further analyses are conducted as required. All analyses are conducted according to the intention-to-treat principle, and will be conducted in SAS v9.3.

### Economic evaluation

The cost of CN on the health system is estimated using standard economic evaluation methodologies. Costings include direct intervention and patient level costs related to hospitalisation and primary care, specialist services and prescribed medications. The costing of health services is based on standard published rates, in particular the Australian Refined Diagnostics Related Group cost weights for hospitalisations, Medicare Benefits Schedule (MBS) charges for medical services and Pharmaceutical Benefits Schedule (PBS) charges for medications. The measure of effect is determined using Quality Adjusted Life Years (QALYs), a measure of life expectancy weighted by health-related quality of life. Average quality of life over the follow-up period for each patient is estimated and weighted by survival to determine QALYs gained post intervention. Incremental cost per QALY gained is calculated between the intervention and control groups.

### Process evaluation

A process evaluation is conducted to describe the organisational setting and context within which CN is developed and implemented. Interviews are carried out with individuals at several levels of program implementation using purposive sampling as follows: a) hospital, Local Health District and NSW Ministry of Health policy and decision makers; b) Nepean Hospital staff in key roles to explore details and processes of CN, as well as barriers and facilitators to its implementation; c) clinical staff with daily indirect involvement with CN to provide broader organizational context; and d) patients in the intervention arm, focusing on their experience of chronic illness and its management as a result of CN. Paired interviews are carried out with patients, their carers, and also the patient’s GP, where possible.

All interviews are recorded and transcribed verbatim. Analysis is thematic, and NVivo v9 is used to assist with data management. Coding is carried out inductively based on the themes that emerge from interviews.

### Ethics

Ethics approval was received from Sydney West Area Health Service Human Research Ethics Committee – Nepean Campus [AU RED: HREC/09/NEPEAN/55], now Nepean Blue Mountains Local Health District Human Research Ethics Committee.

## Discussion

To our knowledge, this is the first study to provide a mixed-method evaluation of the implementation and effectiveness of a care coordination program in Australia that includes a heterologous population and substantial follow-up period. Evidence of any improved patient outcomes and satisfaction due to care coordination are critical, given recent interest by governments. Coordinated care is becoming incorporated at the national policy level in several countries [11,12,17]. The integration and continuity of prevention and care is a priority action area in the National Chronic Disease Strategy of Australia [17]. These policy initiatives must be underscored by a solid evidence-base.

The main strengths of this study are the nature of the CN intervention, the comprehensive process evaluation, and the design of the RCT. The quantitative components of the study utilise a *pragmatic* RCT, allowing heterologous patient groups to be randomised to receive interventions that can be tailored to individual patients based on type, complexity and severity of disease, while still providing statistically meaningful information. The intervention identifies patients in the acute setting with a single point of access, involves GPs, builds capacity within the ED, and places the patient at the centre of decision making using a systems approach to the delivery of health care. A patient-centred focus is essential to ensure the health care and social needs of the population are adequately met [5,7].

The CN process evaluation provides qualitative data to complement outcome measures collected by the trial. If significant changes in patient and health service outcomes are observed, the process evaluation describes the mechanisms underpinning these changes. An account of the implementation of the intervention, and the context of the health system it is conducted within, gives a richer understanding of the intervention and enhances the external validity of the RCT.

The design of the RCT incorporates a large sample size with adequate power to detect the primary outcomes, and a long follow-up period to observe meaningful improvements in patient health outcomes [18,19]. The use of independent random assignment to the intervention, blinding where feasible, and an intention to treat analysis minimises bias.

The main challenges of the intervention are loss to follow-up, heterogeneity of the intervention received between patients, and bias in patient recruitment. Loss to follow-up is minimised by receiving consent at baseline for hospital, medication and death data to be obtained electronically from local and national health databases for the entire follow-up period. Patients are also in regular contact with CN nurses during the period of follow-up to action care plans, however, follow-up data collection is not conducted by CN nurses to minimise bias. The intervention is conducted with consistent protocols to minimise between-patient variability. Care Navigation nurses are trained in these protocols, and intervention delivery checklists are used to record intervention delivery to the patients. To minimise bias in patient recruitment, eligibility criteria are designed to capture all patients who would normally receive CN upon presentation to the emergency department, while still maintaining accuracy and completeness of the data. Efforts are made to accommodate culturally and linguistically diverse patients.

This mixed-methods study informs the generalisability and sustainability of care coordination programs in Australia and internationally. The CN trial aims to build upon these principles to provide a comprehensive coordinated care program for improving health outcomes among patients with chronic illness.

## Abbreviations

EQ-5D: Euroqol quality of life questionnaire; GP: General practitioner; MBS: Medical benefits schedule; OECD: Organisation for economic co-operation and development; PBS: Pharmaceutical benefit scheme; QALY: Quality adjusted life years; RCT: Randomised controlled trial.

## Competing interests

The authors declare that they have no competing interests.

## Authors’ contributions

NP wrote the study protocol, coordinates the RCT, leads study recruitment, and drafted the manuscript. KM substantially revised the manuscript and conducts data analysis for the RCT. PK developed the statistical methods for the study, and provides ongoing study management. TU participated in the design of the study and provides ongoing advice on study management and data interpretation. JG conducts policy and decision-maker interviews and is a senior advisor on the process evaluation. JM designed the process evaluation methodology and is carrying out clinical staff and participant interviews. SJ developed the methods for the economic evaluation and provides ongoing study management. BE conducts data cleaning and analysis of data for the economic evaluation. SB participated in the study design and ongoing study management. KG manages the implementation of the RCT on site. DR and NM are CN nurses and assist NP with recruitment and electronic documentation. CA contributed to the grant writing process. SL conceived of the study, and participated in its design and coordination. All authors revised and approved the final manuscript.

## Pre-publication history

The pre-publication history for this paper can be accessed here:

http://www.biomedcentral.com/1472-6963/13/164/prepub
